# Reproductive decision-making in the context of inheritable conditions that affect appearance: Lived experiences and perspectives

**DOI:** 10.1007/s12687-026-00926-3

**Published:** 2026-07-31

**Authors:** Kerry Montgomery, Diana Harcourt

**Affiliations:** Centre for Appearance Research, School of Social Sciences, College of Health, Science & Society, University of the West of England, Frenchay Campus, Bristol, BS16 1QY UK

**Keywords:** Visible difference, Stigma, Psychological, Genetic counselling, Peer support

## Abstract

Inheritable conditions that affect appearance can influence a range of life experiences shaped by social, emotional, and cultural factors. For individuals with such conditions (e.g., craniofacial conditions), considerations about having children may be further complicated by the possibility of passing on the condition and its associated social challenges. This study explored how the visibility of inheritable conditions that affect appearance influences reproductive decision-making, with particular attention to decisions about having children and the use of reproductive technologies. Semi-structured interviews were carried out with 18 participants (16 female; 2 male) with a range of visible skin conditions (e.g., neurofibromatosis) and craniofacial conditions (e.g., Treacher Collins Syndrome) who had either considered having children, were currently pregnant or trying to conceive, or had had children within the last five years. Interviews were analysed using reflexive thematic analysis and revealed four main themes. In theme one, *“Coping and adaptation in caring for a child with a visible difference”*. parents described an emotional journey from initial distress toward solution-focused coping as they adapted to caring for a child with a visible difference, drawing on their own lived experiences as both a burden and a resource. Theme two, *“Social perceptions*,* stigma*,* and responsibility in reproductive decision-making”* illustrates how societal attitudes, stigma, and memories of negative school experiences shaped reproductive decisions, particularly views on natural conception, IVF-PGT, and termination. Theme three, *“Managing uncertainty and control in reproductive choices”* captured participants’ strategies to manage risk and regain a sense of control through prenatal testing and assisted reproduction, while navigating complex ethical dilemmas. Finally, in theme four, *“Patient-identified needs as a framework for improved support”* participants emphasised the need for unbiased genetic counselling, practical psychosocial support, and opportunities for peer connection. Overall, this study demonstrates that individuals draw on their lived experiences and anticipate stigma their children may face. These social considerations strongly influence choices around having children and reproductive technologies. The findings underline the importance of genetic counselling that explicitly recognises the role of stigma and societal attitudes, enabling practitioners to better support individuals in making informed decisions.

## Introduction

Decisions about whether to have children are widely recognised as complex and deeply personal, shaped by social, emotional, and cultural factors. In healthcare, reproductive decision-making is increasingly recognised as involving more than medical risk, drawing on personal identity, lived experience, and plans and expectations around parenthood. For individuals with genetic conditions, decisions about reproduction may be further complicated by the possibility of passing on the condition to future children and by concerns about the social and psychological implications of inheritance (Stock et al. 2023). Despite this complexity, much research within genetics has focused primarily on reproductive outcomes, rather than on the processes through which individuals make reproductive decisions and the factors that shape them (Gee et al. 2017). Understanding decision-making involves consideration of factors such as access to genetic testing, healthcare support, and attitudes towards in-vitro testing. Exploring how these factors shape reproductive choices is crucial to improving support and guidance for individuals and professionals alike.

Existing research on reproductive decision-making in genetics has largely focused on life-threatening or life-limiting conditions (Gee et al. 2017). Studies indicate that individuals with conditions of lower mortality may feel more conflicted in their decision-making, perhaps because the prognosis is less predictable (Frigon et al. 2022). A study of individuals with cystic fibrosis found that concerns about parental health, caregiving capacity, and the child’s future wellbeing were central to reproductive decision-making. However, these decisions were shaped not only by genetic risk but also by personal identity and the broader social context (Vanhollebeke and Steijvoort 2024).

Visible difference is a broad term used to describe differences in appearance that may result from conditions, injuries, medical treatments, or trauma. These may involve the face, skin, or body and can be congenital, inherited from an affected parent or arising from a de novo variation (e.g., craniofacial conditions including cleft lip and palate and Treacher Collins syndrome, or skin conditions including epidermolysis bullosa and ichthyosis), or acquired (e.g., burn injuries and scarring from injury or surgery). Importantly, the level of distress associated with a visible difference is not always linked to how visible or severe the condition appears (Moss et al. 2014). While the medical features of these conditions can vary widely, their visible nature can have a significant impact on psychological wellbeing and quality of life (Fournier et al. 2023; Stock et al. 2023; Zucchelli et al. 2023). For individuals with inheritable conditions that affect appearance, these psychosocial challenges can become particularly salient when considering parenthood and the possibility of passing the condition on to a child (Stock et al. 2023). Although medical management is often central to care, the social and emotional challenges associated with visible difference can play a significant role in shaping reproductive decision-making. These influences are not always explicitly recognised or addressed within healthcare where discussions may prioritise medical risk over lived experience and psychosocial concerns. As a result, the ways in which visible difference affects decisions about having children, and the implications this has for the support provided by healthcare professionals, remain under-examined. Greater understanding of these challenges is needed to ensure that care appropriately reflects the factors influencing individuals’ reproductive choices.

Research suggests that the psychosocial outcomes of conditions affecting appearance are similar whether the condition is congenital or acquired, indicating that early onset does not necessarily offer an advantage (Zucchelli et al. 2023). As a result, some adults may struggle with adjustment throughout their lives, which may influence reproductive decision-making, though the extent of this influence remains underexplored. Studies of genetic conditions that affect appearance show that young adults with cleft lip and/or palate often view psychosocial challenges as equally, if not more, significant than medical issues when considering having children (Stock and Rumsey 2015). In Neurofibromatosis, prospective parents frequently reflected on their own difficult social experiences, particularly from childhood (Barke et al. 2016), perhaps because these memories felt most salient or because prospective parents are particularly concerned about the anticipated quality of life in their child’s early years.

Individuals with craniosynostosis have been reported to be less likely than the general population to have children (75% vs. 18%), and content analysis indicates varied motivations within this group. Some participants felt they did not want children to avoid potential suffering for future generations, while others believed they could cope with parenthood given adequate support (Stock et al. 2023). However, it is important to extend this line of research to understand what drives these differing perspectives and to identify what forms of support would be most helpful.

Another important area of inquiry concerns the use of Assisted Reproductive Technologies (ART e.g., Pre-Implantation Genetic Testing with In-Vitro Fertilisation; IVF PGT) and prenatal testing. Gee et al. (2017) conducted a scoping review of studies involving parents of children with rare genetic conditions and found that parental perceptions of their ability to cope with an affected child were central to decisions about using reproductive technologies and used scenario-based thinking when making reproductive decisions. There is a need to explore decision-making around ART in people with an inheritable condition that affects appearance the current study focuses on individuals with conditions included on the Human Fertilisation and Embryology Authority’s (HFEA) list of approved conditions for ART. As Gee et al. (2017) emphasise, there is a need to support parents when they are making reproductive decisions and a need to be aware of how and when to provide support.

Reproductive decision-making is complex and shaped by a range of personal, medical, and social influences, yet there is limited understanding of how these factors are experienced by individuals with inheritable conditions that affect appearance. This study aims to explore the experiences of individuals with an inheritable condition that affects appearance to understand how the visible nature of the condition intersects with genetic, medical and societal factors to influence decisions around having children and the use of assisted reproductive technologies (ART), to inform both prenatal and postnatal support.

## Method

### Design

A qualitative design using semi-structured interviews was chosen to give participants an opportunity to describe their experiences in their own words and to explore how they make sense of complex and sensitive issues related to reproductive decision-making. This approach enabled a detailed understanding of personal perspectives and decision-making processes. Qualitative research is widely recognised as a valuable way of gaining in-depth insight into lived experience and understanding how individuals’ accounts are shaped by their social and cultural environments (Camic 2021). Despite this, healthcare research has often prioritised quantitative methods, meaning qualitative studies are frequently expected to demonstrate their rigour and clinical relevance more explicitly (Wainstein et al., 2023). In genetic counselling research, where findings often aim to inform clinical conversations, service delivery, and patient support, qualitative approaches are particularly important for capturing perspectives that directly influence practice.

This study was developed in collaboration with a Research Advisory Group comprising six individuals with an inheritable visible difference. The group was involved to ensure the research was grounded in lived experience and addressed issues that were meaningful, relevant, and sensitively framed for individuals making reproductive decisions. In addition, we consulted seven representatives from UK based charities supporting a range of conditions that affect appearance to better understand the nature of the support they provide around reproductive decision-making. Insights from these consultations informed key aspects of the study design, the focus of the research questions, recruitment materials and the interview schedule. Ethical approval for the study was granted by the Research Ethics Committee at the University of the West of England.

### Recruitment

Participants were recruited using purposive sampling between May and October 2024 via social media and UK-based the charities working with individuals with inheritable visible differences (see acknowledgements). Although many conditions affect appearance, recruitment was conducted through charities within the Appearance Collective, a group of charities supporting individuals with conditions that affect appearance, and via the Centre for Appearance Research participant pool, which targeted specific conditions. The study advert contained brief details of the research including the study aims, inclusion criteria and contact information of the researcher. The advert also contained a link to an online information sheet, an online consent form and a short online demographic questionnaire. The questionnaire collected information including condition, age, gender, marital status and whether they have children. Eligible participants took part in a one-on-one, semi-structured interview conducted online through Microsoft Teams. Each interview lasted approximately 50–90 min. To thank participants for their time they were offered the choice of a £25 online shopping voucher or a donation of £25 to any charity of their choice.

### Inclusion criteria

Participants were eligible if they were aged 18 or older, living in the UK, and had a visible difference that could be inherited by their biological children. To explore conditions relevant to assisted reproductive technologies, we recruited individuals whose conditions appear on the Human Fertilisation and Embryology Authority (HFEA) list of conditions eligible for IVF with pre-implantation genetic testing (IVF-PGT), ensuring eligibility for genetic testing and ART.

Inclusion criteria also required participants to have considered starting a family, be pregnant or currently trying to conceive, have had children within the past five years, or have decided not to have children. The cut-off point of five years for the age of participants’ children was chosen to account for advances in medical and genetic technologies. Couples with older children may not have had access to the same treatments or information, which could influence their decision-making. This cut-off was agreed upon following consultation with genetic counsellors and the lived experience advisory group. Exclusion criteria included conditions for which there is no known genetic link that can be tested, and individuals who had not yet considered having children.

### Analysis

Interviews were recorded and transcribed using Microsoft Teams’ transcription feature, with the researcher reviewing and editing the transcripts for accuracy. Interviews lasted between 50 and 90 min. All transcripts were pseudonymised and any potentially identifiable data (e.g., hospital attended, geographic location) was removed. The interview guide was developed collaboratively with the Research Advisory Group and charity representatives to ensure sensitivity and relevance, and no pilot interviews were conducted, as questions were refined through this process prior to data collection. Given the paucity of research in this area an exploratory in-depth inductive qualitative approach, was used to understand individuals’ experiences.

Interviews were analysed using Reflexive Thematic Analysis (RTA), guided by Braun and Clarke’s (2023) six-step framework. One reason for choosing Reflexive Thematic Analysis (RTA) is the recognition of the researcher’s active role in identifying patterns and meaning in the data, recognising the researcher as part of the meaning-making process rather than as a threat to objectivity (Braun and Clarke 2019). Familiarisation occurred through transcription and anonymisation, followed by initial inductive coding in NVivo (version 14). Coding was led by the first author with codes developed and refined iteratively across transcripts. Themes were developed through an ongoing process of reviewing, organising, and refining codes to identify patterns of shared meaning across the dataset. Developing themes and analytic decisions were discussed regularly with the second author. These reflexive discussions were used to question interpretations, clarify the boundaries between themes and subthemes, and ensure coherence, rather than to achieve consensus coding, in line with the principles of RTA (Braun et al. 2023).

### Positionality

The first author has a genetic condition that affects appearance and has accessed genetic testing in the past. The second author has surgical scarring that is not usually visible to other people and over 25 years’ experience of psychosocial research around visible difference. It is important to acknowledge the authors’ experiences of visible difference, particularly the first author’s position as a ‘me-researcher’ (Bogart 2024), and the potential influence this may have had on data collection and analysis, including participants’ willingness to share their experiences and the interpretation of the data’s meaning. Through ongoing reflexive dialogue with the second author, the researcher’s personal experience helped shape how participants’ accounts were approached and interpreted, while remaining attentive to differences between the researcher and participants. When acknowledged and reflected upon, lived experience can help identify gaps in research and draw attention to issues that might otherwise remain unexplored (Bogart 2024).

## Results

Participant details (see Tables 1 and 2) are presented; however, demographic and clinical factors have been reported separately and are not linked to individual participants to maintain confidentiality.

**Table 1:** Participant demographics

Demographics	Category	*N*
Gender	Male	2
	Female	16
Age	18–24 years	1
	25–34 years	10
	35–44 years	4
	45–54 years	3
Ethnicity	White British	16
	Asian/British Asian	2

**Table 2:** Demographic characteristics and parental status of participants

Participant characteristics	Category	*N*
Condition	Genetic Skin conditions:	14
	Neurofibromatosis type 1	7
	Ichthyosis	3
	Epidermolysis Bullosa	2
	Ectodermal Dysplasia	2
	Craniofacial conditions	4
	Cleft lip and palate	1
	Treacher Collins Syndrome	2
	Crouzon Syndrome	1
Decisions regarding reproduction	Decided not to have children	4
	Considering having children	3
	Currently undergoing IVF PGT	2
	Currently pregnant (via IVF PGT)	2
	Currently pregnant (non-IVF PGT)	1
Participants who have children	Had a baby via IVF PGT	1
	Had a baby (non-IVF PGT)	6
	Child affected	4
	Child unaffected	2
	Unknown	1

Table 3 presents the themes that capture participants’ experiences of reproductive decision-making and their considerations regarding having children. Four key themes were identified theme 1: *Coping and adaptation in caring for a child with a visible difference*, theme 2: *Social perceptions*,* stigma*,* and responsibility in reproductive decision-making*, theme 3: *Managing uncertainty and control in reproductive choices.*

**Table 3:** Themes and subthemes

Theme	Subthemes
Theme 1: *Coping and adaptation in caring for a child with a visible difference*	1.1 *Finding out: Emotional responses to diagnosis and early adjustment*
	1.2 *Parental coping as a dynamic process over time*
	1.3 *Parenthood*,* responsibility*,* and anticipated stigma in the context of visible difference*
Theme 2: Social perceptions, stigma, and responsibility in reproductive decision-making	2.1 *Preventing suffering as a central motivation*
	2.2 *Emotional responses and ethical dilemma around termination*
	2.3 *Dealing with social pressures and judgements around decisions to have children*
	2.4 *Intergenerational perspectives and the limits of parental influence*
Theme 3: Managing uncertainty and control in reproductive choices	3.1 *Influence of perceived severity on decision-making*
	3.2 *Experiences of genetic counselling: navigating facts and feelings*
	3.3 *Your body your decision. Reproductive agency and partner involvement*
	3.4 *More than making a baby: IVF and PGT as a way to take control and find hope*
Theme 4: Patient-identified needs as a framework for improved support	4.1 *Barriers and pathways to professional support*
	4.2 *Peer connection and community as a source of strength*
	4.3 *Unmet needs and reflections on helpful support*

The majority of participants (*n* = 14) had congenital skin conditions, with Neurofibromatosis being the most common. Including these varied perspectives was essential to capturing the full complexity of reproductive decision-making. Participants described wide variation in the perceived severity of their own condition, ranging from mild to “severe.” For some, the condition had minimal medical or psychosocial impact; however, for others, the associated physical and psychosocial challenges significantly affected everyday life. There were clear differences in access to healthcare across conditions. Participants with skin conditions including Neurofibromatosis and Ichthyosis continued to use specialist NHS services, while those with craniofacial conditions had been discharged in their late teens and no longer had access to dedicated teams. This shaped access to genetic testing and counselling. For those with skin conditions, family planning was usually discussed within specialist clinics, whereas participants with craniofacial conditions were largely unaware of available reproductive support and only sought general genetics services when they began considering having children. These differences likely reflect clinical practices at the time of discharge (around 10–20 years earlier). Including a range of conditions enabled comparisons of how visibility, severity, and service access influenced reproductive decisions and any shared and condition-specific patterns. Four overarching themes were identified, each comprising subthemes that further describe the key factors influencing reproductive decision-making among individuals with genetic conditions that affect appearance.

## Theme 1: Coping and adaptation in caring for a child with a visible difference

Theme 1 aims to capture participants perceptions of their initial responses to their child’s diagnosis and describes how they learned to cope over time. The theme also describes participants perceptions of parenting alongside the challenges of navigating social interactions related to their child’s appearance.

### 1.2 Finding Out: Emotional responses to diagnosis and early adjustment

Participants described feelings of guilt when their child inherited their condition. For some, this guilt emerged immediately at birth, reflecting the emotional impact of being confronted with the visible reality of the condition. Nadia described feeling shocked and distressed following the birth of her daughter, highlighting a potentially traumatic experience for both herself and her partner. Her account illustrates how responsibility for genetic transmission can become internalised, with parents attributing blame to themselves at a highly vulnerable moment.

*“I remember the doctor came over; he obviously couldn’t confirm it because he can’t confirm it until they’ve got the genetic testing back. I remember him just saying she does have EB. Well*,* that was it. I was crying. I was saying*,* it’s all my fault. This is my fault. I remember apologising to my partner. I remember looking round the room and everyone was crying and the anaesthetist was crying*,* everyone was tearful” Nadia*.

Casey found out through prenatal testing and this initial shock led to feelings of not wanting to continue the pregnancy. This shock may be in part explained by Casey reporting being unaware of the chances of a child inheriting her condition being higher than what she had understood.

*“I found it very hard to go through that*,* got to the 20 weeks and went in and then all of a sudden*,* the sonographer just went quiet and I think I knew like I just sort of saw something and I thought is that and it was the worst thing I could have possibly heard*,* no one could console me and truthfully*,* I just walked out and I just said no*,* No. This baby is not to be. I don’t want it. You need to help me make those steps” Casey*.

Casey later reflected on how she worked through these feelings and chose to continue the pregnancy. Receiving the diagnosis at 20 weeks’ gestation provided time to access psychological support and begin processing her feelings about her appearance and her worries about her child. This period of adjustment appeared to facilitate a shift from initial distress and uncertainty towards greater acceptance and preparedness for birth. Casey’s account highlights how access to support following a prenatal diagnosis can help parents navigate complex emotional responses.

### 1.2 Parental coping as a dynamic process over time

One challenge of caring for a child with the same condition was that it could bring up difficult past experiences. To manage these emotions and the guilt associated with passing on the condition, some participants adopted avoidant coping strategies, such as delegating aspects of care to a partner or mentally distancing themselves. However, these strategies could create further emotional conflict, including feelings of not being “motherly.”

*“The hardest part for me is that I still experience a lot of trauma when doing her skin care. My partner actually does it more than I do because*,* when I try*,* I either feel guilty or*,* if I try not to feel guilty*,* I have to completely dissociate. Then I feel guilty about that too*,* because it makes me feel like I’m not being a good mother by distancing myself from the situation.”* Nadia.

Although participants initially described feelings of guilt when learning their child had inherited their condition, many also spoke about a shift in mindset after their child was born. Over time, they began to focus on what they could take from the experience, reframing challenges as opportunities for resilience and growth. As Georgia reflected:

*“I think you know these little bumps in the road just help to build resilience*,* don’t they? And I feel like I’ve come out the other side of this far more resilient than I ever was. So you know*,* you’ve got to try and take be good out of a situation*,* haven’t you?” Georgia*.

In addition, participants described using solution-focused coping strategies to manage their child’s condition, from ensuring they had all the practical items they needed and ensuring professionals involved in the child’s care understood their needs.

*“Everything we bought all the clothing we bought*,* everything we bought*,* we made sure was bought with the idea of it needs to be skin friendly even the car seat.” Nadia*.

### 1.3 Parenthood, responsibility, and anticipated stigma in the context of visible difference

A child’s visible difference often acted as an emotional trigger, re-activating parents’ own memories of growing up looking different. These recollections of exclusion, stigma, and social vulnerability shaped how parents understood their role and responsibility, frequently giving rise to feelings of guilt about having passed the condition on. Rather than reflecting distress about the medical aspects of the condition, these responses were rooted in parents’ anticipation of the social challenges their child might face, based on their own lived experience.

*“It’s stirred up again a lot of the feelings for me of how I felt when I was at school and feeling different and not accepted and being excluded and I don’t want him to go through that. What can I do to try and really make sure he doesn’t have the same experience as I had?” Chloe*.

For Casey, fears of judgement from other people would make judgements about her decision to have children, as Casey’s experience illustrates this could lead to heightened self-consciousness and anticipatory stigma.

*“I didn’t want people to see me because as soon as he’s born they’re going to see me and then I have to acknowledge something has happened to me too…. surely they’re all gonna be asking me questions like why would you do that to your child? so a lot of projection and a lot of speculation based on the world that I’ve experienced” Casey*.

Parenting a child with a visible difference can prompt parents to reflect on difficult emotions linked to their own past experiences, which may not have been revisited or fully considered before becoming a parent. As a result, parenthood can present challenges as individuals navigate wider social perceptions of visible difference and consider how these perceptions relate both to themselves as parents and to their child. These issues around social perceptions and external influences are explored in greater depth in Theme Two.

## Theme 2: Social perceptions, stigma, and responsibility in reproductive decision-making

Theme two captures how social perceptions and feelings of responsibility shaped reproductive decision-making, alongside the influence of intergenerational views, particularly parents’ perspectives. Participants past experiences of stigma, especially in school, played a central role, often motivating their desire to protect their child from similar challenges. Family-planning decisions were not made in isolation, participants views about looking different, using reproductive technologies (e.g., IVF-PGT), and ending a pregnancy interacted and influenced how much social pressure they felt and their worries about being judged by others.

### Theme 2. Preventing suffering as a central motivation

While participants mentioned physical symptoms, they more often expressed concerns about the potential psychological distress a child might experience, particularly in relation to school. Participants worried about prospective children having negative school experiences, with these concerns reflecting their own lived experiences. In this way, parents appeared to project their past experiences onto their children, assuming similar social challenges were likely to occur, rather than holding the view that, although this had happened to them, their child’s experience might be different. Early school events appeared particularly salient, shaping participants’ emotional responses into adulthood and influencing anxieties about future children.

*“I could never see my child going into school*,* feeling vulnerable*,* feeling as if they’re going to be stared at or looked at or pointed or not wanted. I just couldn’t bear that thought. I couldn’t do that*,* even imagining what I was going through when I was in school*,* it makes me emotional.” Abel*.

Max also noted limited understanding from teachers, suggesting gaps in perceived social support.”

*“You don’t want your children to go through the same things necessarily. School life was quite difficult in primary school especially*,* back then*,* the teacher didn’t really do anything*,* they just say ignore it*,* but it’s not as easy as that.” Max*.

In addition to drawing on past social experiences, participants also reflected on their own medical histories and experiences of hospital settings. While many parents expressed a desire to protect future children from difficult social experiences, some participants were concerned about the prospect of re-living the medical side of things. These concerns were not solely about practical burden, but about the cumulative emotional impact of having to go back into hospitals with their child.

*“I’ve had my time with hospitals. I’ve done that*,* I still have appointments to go to*,* I’m going to have to potentially live through that again*,* which of course you would do for your child*,* but I think I realised do you know what? I’m not going to have any children. I don’t want to have to go through that again with the hospitals.” Anna*.

Anna’s account illustrates how her own medical experiences influenced her decision-making, and there is a sense of having reached her limit with hospitals, feeling that she had already spent much of her life engaged with healthcare, Anna expressed a strong desire not to face this experience again through parenthood.

### Theme 2.2. Emotional responses and ethical dilemmas around termination

Discussions around prenatal testing and termination can bring up difficult ethical dilemmas for people as feelings are often shaped by cultural, religious and social beliefs. Interestingly, while all participants identified as pro-choice when discussing termination of pregnancy, the majority (16 out of 18) stated that they would not choose to terminate a pregnancy if the prospective child had their condition. As a result, prenatal testing was ruled out due to the associated risk of miscarriage and that termination would not be considered regardless of the outcome. This theme captures an intensely personal and potentially taboo subject that many individuals may find difficult to talk about openly. One participant, Casey conveys a moral conflict, in which the decision to terminate a pregnancy became inseparable from her own sense of identity and self-worth. This excerpt highlights how reproductive decision-making can be shaped by identity, self-acceptance, and personal values, and how these factors interacted to inform views of reproductive decisions.

*“Truthfully if I aborted this baby*,* essentially*,* I’d just be you know*,* why haven’t I aborted my own life? like*,* essentially killing my reflection is what I was doing*,* and trying to save my own skin and that was really the crux of it. I knew I couldn’t do that. I knew that was wrong for me.” Casey*.

### Theme 2.3 Dealing with social pressures and judgement around decisions to have children

This subtheme captures how perceived social pressures and judgments surrounding having a child with a visible difference shaped participants’ sense of self and reproductive decision-making. Participants described a tension between broader societal expectations that women should have children and a perceived disapproval of people with visible differences having children with the same condition. This reveals a conflicting social dynamic in which expectations to have children coexist with anticipated judgment about difference. Kelly articulated this perceived judgment clearly:

*“Certain people in society don’t agree that people with disabilities go on to have children with disabilities. I think it’s that kind of*,* ‘am I going to get a backlash from people being like how dare you or how selfish that you’ve wanted to have a child” Kelly*.

Underpinning these concerns was a broader perception that visible difference itself is viewed negatively within society, as illustrated by Lexi:

*“Difference is a bad thing*,* I think in the world so if you have a 50% chance of having a child who is very different*,* that’s a bit dramatic*,* but it’s going to have an impact on how they’re able to interact with the world*,* I think.”* Lexi.

Lexi’s concerns focus less on medical issues and more on how societal attitudes may affect her child’s ability to be accepted and engage in everyday activities. This highlights how decisions about having children are shaped by expectations of social judgement and anticipation of social exclusion.

### Theme 2.4 Intergenerational perspectives and the limits of parental influence

Participants perceived their parents as experiencing guilt, regardless of whether the condition was inherited or arose de novo. Participants discussed how their parents’ own experiences of parenting a child with a visible difference shaped their views on prospective grandchildren, with some offering unconditional support and others expressing strong opinions about not wanting their child to pass the condition on to a grandchild. Parents’ views were often clearly expressed and grounded in their own experiences of raising a child with a visible difference; however, these views did not always shape reproductive decisions in predictable ways. While some parents or grandparents offered strong emotional support, this did not necessarily translate into individuals feeling more able or willing to have children.

*“I remember when I was a teenager when I said that I wanted to have a baby my mum said to me you couldn’t have a baby with EB because that would just destroy us all*,* that would be really difficult and blah*,* blah*,* blah and I remember saying to her would you have terminated me if you knew? I remember she was taken back” Nadia*.

Nadia’s account illustrates how parental concern was framed around the perceived emotional and practical impact the child having the condition would have on the wider family. Interestingly, parents expressing strong views about not passing on the condition did not necessarily discourage participants from considering parenthood; however, parenting styles did appear to influence how participants imagined and approached future parenting styles.

*“In general me and my parents aren’t great at talking about many things*,* but I feel my diagnosis was probably the benchmark for that because that changed how I communicated with people. I’ve never heard ‘you look pretty’ or ‘you look beautiful’*,* I don’t recall ever hearing that…I cannot just pretend that he (son) was born with a diagnosis that does not exist because that would be so ludicrous and although I’m spotlighting it on him*,* actually all children need to grow up feeling confident and having self-worth and all of those things. It’s going to be very different in comparison (to her own upbringing) for the very reason that I want all of them (children) to have somewhere they can come and speak*,* whether it’s about that (condition) or whether it’s about something else.” Casey*.

Casey talked about how her own upbringing and diagnosis shaped communication within her family, particularly around appearance and her feelings of self-worth. As a result of her parents communication style and the impact this had on her emotionally Casey expressed a strong commitment to creating an open, supportive environment for her children. Her approach to parenting reflects an intentional effort to avoid repeating her childhood experiences, ensuring her children feel able to talk openly and develop confidence and self-worth.

## Theme 3: Managing uncertainty and control in reproductive choices

Theme three illustrates how medical factors shaped participants’ reproductive decision-making, particularly in relation to prenatal testing and reproductive technologies. Participants’ interpretations of medical information about inheritance risk and condition severity, their interactions with healthcare professionals, and their understanding of what reproductive technologies could offer all played a role in shaping decisions. These medical considerations did not operate in isolation but were shaped by participants’ feelings about having a child with a visible difference. The theme also highlights the role of partners, with most participants describing how their partners viewed reproductive decisions as ultimately resting with the woman, given that any procedures would directly involve her body. While prenatal testing was often framed as a means of gaining information about a child’s condition, it also raised ethical concerns, particularly in relation to termination. For some participants, even small procedural risks associated with prenatal testing were seen as unacceptable, reinforcing decisions to avoid this pathway.

### Theme 3.1 Influence of perceived severity on decision-making

While some participants assumed their child would inherit their condition in a similar way, others acknowledged the possibility of greater severity which was their main concern. For Alexa, the unpredictability of the condition and the possibility that a child might develop a more severe form played a central role in her decision-making. Her account suggests that this concern was shaped both by her awareness of others’ experiences and by her own personality, particularly her reluctance to take risks and desire to protect a future child.

*“I had my own personal experience and I also had that of knowing and reading about other people’s experience with the condition and I knew the unpredictability of what severity a baby might have with and what the possibilities may or may not be. I’ve never really been much of a risk taker*,* unfortunately and I think if I can prevent some sort of*,* I don’t want to say suffering*,* but if I can prevent some challenges to my future children*,* then that’s what I wanted to do for me” Alexa*.

Chloe described feeling able to draw on her own knowledge of the condition to support her child; however, she continued to hold concerns about its physical impact and the potential effects on her child’s appearance.

*“I was worried that my son would get a more severe case than I have because mine is very mild and it can be a lot worse. That was my main worry because I could tell him how to cope with what I’ve got but with the other types of my condition you have to bathe twice a day to get the skin off and you’re just read raw and it just looks horrible.” Chloe*.

### Theme 3.2. Experiences of genetic counselling: navigating facts and feelings

For some participants, the condition was inherited, while in others it arose from a de novo variation and as a result knowledge about the condition and understanding of genetic testing and genetic counselling varied. Nadia described how engaging with a genetic counsellor early helped her understand her options and make informed decisions. This knowledge influenced the precautions she and her partner took to avoid an unplanned pregnancy, showing how engaging with genetic professionals early can help people to make informed choices around sexual health and family planning. She explained,

*“I knew very early what my options were and what that meant going forward. So then when I did meet my partner*,* we were very then careful to not get pregnant because we were conscious of an accidental pregnancy could really change a lot*,* well*,* having a baby changes a lot of things anyway*,* but having an unwell baby would change a massive amount of things in your life*,* and putting that risk on I was really hyper aware of it so we were*,* you know*,* very careful not to. I benefitted from having early input from a genetic counsellor.” Nadia*.

Whilst some participants reported positive experiences of genetic counselling and found it helpful others described mixed or less positive experiences, highlighting variation in how genetic counselling was perceived. Laura felt that, rather than providing an unbiased perspective the genetic counsellor and her doctor were encouraging her to consider assisted pregnancy options to reduce the risk of passing on her condition.

*“I feel like they (genetic counsellor) had an opinion and I feel like their opinion was that if we can stop the child from having NF and we can help you select an embryo where the NF gene is not there then that is the better option*,* and that was from the very blunt lady*,* who said it very matter of factly. The emphasis was on the IVF and the genetic testing as opposed to if you do have a kid with NF*,* this is how we can help support. It was very much*,* this is how we can help you stop a kid from having NF.” Laura*.

While some participants perceived that genetic counsellors’ personal views about reproductive options influenced discussions, it is also important to consider the possibility of an implicit assumption that engaging with genetic counselling signals openness to prevention-focused options, such as IVF-PGT. However, Laura’s experience highlights the importance of presenting all reproductive options in an equally balanced way, particularly given that genetic counselling often involves deeply personal and sensitive considerations about life, identity, and future family choices.

### Theme 3.3 More than making a baby: IVF and PGT to take control and find hope

IVF with preimplantation genetic testing (PGT) allows embryos to be screened for inherited conditions before transfer. Participants expressed mixed views - some saw it as the only way to prevent passing on their condition allowing them to take control and giving them hope for having a family, while others held ethical or personal reservations. Laura’s reflections highlight the difficult decisions faced by people living with inherited conditions when considering their reproductive options. Although she recognised the potential benefits of IVF-PGT in reducing the chance of passing on her condition, she was also concerned about what this choice might mean in relation to how people with the condition are viewed. Her account suggests that reproductive decision-making is not only about preventing future health challenges but also about reconciling personal experiences of living with a condition that forms part of one’s identity.

*“I’m fully pro-choice*,* but I also think that if you’re gonna discriminate against my baby who has Neurofibromatosis where do you stop that discrimination going on to Dolly the sheep and designer babies? Where do you stop until you have that perfect child?” Laura*.

For Chloe the idea of selecting unaffected embryos felt like it was disregarding her identity and aimed to eliminate the condition. Although Chloe didn’t want a child to suffer, the idea of selecting embryos was too uncomfortable and she decided this was not an option.

*“I wouldn’t want a child to go through what I went through but straight away as soon as they said they would get rid of those (affected) embryos I was like*,* that’s almost saying that people with my condition shouldn’t be here. We’ll just disregard that person*,* even though at embryo stage it’s not necessarily a person. It just didn’t sit right with me because just saying people like you*,* getting rid of people like you*,* so that was kind of why I then moved away from that (IVF-PGT) as an option.” Chloe*.

For other participants IVF-PGT offered hope and worries around the child experiencing physical and social challenges outweighed anxiety about the IVF-PGT process. For Georgina, IVF-PGT was the only option she felt would enable her to have children.

*“I knew for us if IVF hadn’t been available we wouldn’t have had an opportunity to have children. It really was a game changer and it all fell together at the right time” Georgia*.

In summary, decisions about IVF-PGT are complex and involve reflections on personal identity and what choosing IVF-PGT might signal about the value of lives lived with the condition. Importantly, the ethical and moral questions associated with IVF-PGT did not resolve once a decision had been made. Instead, these considerations persisted throughout different stages of the process, with each step, such as embryo selection, bringing new dilemmas and prompting ongoing reflection. However, despite the significant ethical and emotional complexity involved, IVF-PGT also provided hope for some families who felt they had no other alternative options for having a child.

### Theme 3.4 Your body, your decision: Reproductive agency and partner involvement

Female participants described partners as supportive but often deferring to them in reproductive decision-making. Some noted that this may have led partners to downplay their own wishes. Participants also felt that partners held a different, often less challenging view of the condition, shaped by seeing it through the lens of an adult who had adapted and whom they loved, which in turn influenced decisions about having children.

*“He just wanted to have a baby because the only skin he knows is the skin I have now and all I have to do is cream in the morning*,* cream at night and have a bath once a week. That’s it*,* he’s seen the photos of me as a baby*,* but he just doesn’t have that same knowledge of it.” Nancy*.

Raising the subject of having children with a partner was difficult but important in the early stages of the relationship. Sally explained that when she needed to discuss this with her partner, her difficulty talking about her condition led her to choose to write a letter instead.

*“I do really struggle so I wrote him a letter… to tell him I’d got NF because I just it’s just one of those things that I really struggle with because I just think people are going to think oh I don’t know. It’s just I’m not a very confident person*,* not many people know I’ve got NF.” Sally*.

Talking to a partner about a genetic condition can be a struggle. A partner might not recognise a condition as genetic, and because many conditions are rare, they may not know what the condition is or understand its genetic implications. As a result, this may still require discussion and explanation, including whether the condition can be inherited and the probability of this.

## Theme 4. Patient-identified needs as a framework for improved support

Exploring participants’ perceptions of support helps to identify what is currently available, what further support might be needed and how it might be delivered. Participants felt that speaking to others in similar situations was a valuable source of support when navigating decisions about having children and during the early years of their child’s life, while also recognising unmet needs in this area. Participants consistently described a lack of support specifically tailored to parents who had the condition themselves, including recognition of their own experiences and knowledge of the condition. This highlighted a gap between general services and the distinct needs of those navigating parenthood alongside their lived experience of visible difference.

### Theme 4.1. Barriers and pathways to professional help

On the whole participants who accessed genetic counselling generally described the experience as informative and supportive. However, some participants perceived that healthcare professionals were more inclined to emphasise options that reduced the likelihood of having an affected child. Alongside this, several participants reported practical barriers to accessing genetic services, including long waiting times, uncertainty around referral pathways and eligibility for genetic testing. Participants also reflected on changes in clinical practice over time; whereas conditions affecting appearance, such as some craniofacial conditions, were previously diagnosed primarily through clinical assessment, there is now an increasing emphasis on early genetic testing and diagnosis.

*“I went to my local GP and said I need to be referred to genetics to get my genetic testing done and my GP at that time didn’t really understand genetics. He didn’t understand why I didn’t have my genetic testing done but it was 25 years ago*,* he didn’t understand the technology wasn’t available and he didn’t understand why I wanted to have testing so he pretty much said no*,* I’m not getting referred. It’s going to cost too much.” Alexa*.

Long waiting lists for genetic services and counselling through IVF led participants to access therapy privately suggesting that the demand for psychological support outweighs the available provision.

*“I did get in touch with a therapist again at that time*,* so fortunately the IVF service does provide a few sessions of counselling*,* but again*,* there’s a waiting list for that*,* so I did go privately for the therapy that I needed” Alexa*.

If people are engaging with private support service access figures may underestimate levels of need, as NHS statistics do not capture individuals who seek reproductive counselling related support through private providers. This suggests that existing data may not fully reflect the demand for support or the experiences of those navigating care outside NHS pathways.

### Theme 4.2. Peer connection and community as a source of strength

Participants found connecting with others who shared the same condition to be a particularly valuable source of support. Peer connections were formed through online spaces and in-person groups and were described as helping to normalise experiences, reduce anxiety, and provide reassurance based on shared lived experience.

*“That (charity) Facebook community is amazing*,* the shared experiences*,* and seeing people*,* for example*,* when I found out about the glioma*,* I searched in the chat thing and there were people mentioning the glioma being like oh*,* we’re not worried about it*,* it’s not grown because it’s optic nerve glioma sounds so scary.” Laura*.

Laura’s account illustrates how online peer communities enabled her to make sense of potentially frightening medical information by drawing on the experiences of others who had lived with the condition over time. Abel reflected on the role of peer support while navigating the IVF-PGT pathway, and the distinction between speaking with someone who had pursued IVF due to infertility and someone who had used IVF-PGT because of a genetic condition. While recognising the complexity of this distinction, he described a sense of tension between these experiences, which shaped how he understood peer support.

*“I think it’s slightly different*,* first of all*,* those people are going through a different experience*,* they’re going through an experience where they aren’t able to have a child for an XY or Z reason*,* whereas we are going through this*,* there might be a bit of a*,* not conflict*,* that’s not the right word*,* but they might feel as if*,* oh*,* your appointment is taking away*,* do you get what I mean? it’s hard to explain it just adds another layer of complexity I think*,* for people with visible differences because it’s just there*,* it’s in your in your face and it’s always*,* you can never hide it.” Abel*.

For Abel, having a visible difference appeared to add an additional layer of complexity to the IVF-PGT process making it difficult to fully relate to those whose IVF journey was driven by infertility alone. This suggests that peer support is most effective when it reflects both the practical pathway (such as IVF-PGT) and the experiences tied to visible difference, reinforcing the importance of condition-specific and context-sensitive peer connections.

### Theme 4.3 Unmet needs and reflections on helpful support

Participants were asked to describe any support they received which was helpful whilst also acknowledging any unmet needs and how professionals could bridge these gaps. This includes ensuring that people with conditions that affect appearance who are considering having children are aware of the support available to them. However, as Sally reflected, it is equally important that people who choose not to have children are also supported around this decision. Health care professionals should be aware of services who can provide counselling and psychological support to signpost individuals who would benefit.

*“Maybe just a bit more counselling about things even when you have decided not to have a child ‘cos I do think it affects you when you’re desperate for a child and then you have to decide on your own and not to go on to have a child and just you get sort of just left….so maybe if you decide not to have children you could see a counsellor” Sally*.

Casey accessed support through a specialist team and described how having space to talk about her experiences was helpful, noting that approaches such as radical acceptance and Dialectical Behaviour Therapy (DBT) were particularly helpful during her pregnancy.

“*The tools that you use*,* like a radical acceptance with a DBT tool*,* was for me the most beneficial thing that I’ve taken forward and just being able to talk about it*,* I think that one hour a week having somebody to talk to*,* it was massive. That was key and it was me*,* it was my experience” Casey*.

While specific therapeutic approaches were beneficial, Casey highlighted that simply having space to talk about her own feelings related to visible difference was particularly valuable. Having this kind of support before a child is born may help individuals work through difficult feelings about their own visible difference and be in a more positive place at the time of birth, which may, in turn, support the early parent–child relationship.

Participants discussed that more information and resources to support reproductive decision-making would be helpful. Practical resources were seen as especially valuable, including visual materials such as videos and guidance on navigating everyday challenges, for example how to talk to employers about undergoing IVF-PGT.

*“I think speaking to other people who have either made the decision either way or gone through a similar thought process and how they came to the decisions like what was their crux of why they achieved it*,* and if there any books on it or anything like videos*,* just to really see how they made their decision” Jess*.

Overall, there is a need for greater support when making reproductive decisions, alongside a desire to hear the experiences of others with visible difference. Both formal therapeutic support and more informal peer support were seen as valuable. However, the way support is delivered needs consideration, as not everyone can access face-to-face support due to time or geographical constraints, particularly given the rarity of these conditions therefore professionals should consider alternative formats, such as online resources, videos, or written materials.

## Discussion

The current study explored reproductive decision making among individuals with inheritable conditions that affect appearance. The focus on appearance-related conditions was to understand how the visibility of a condition influenced decisions about having children and/or using reproductive technologies. Consistent with previous research, the findings highlight the importance of social experiences and personal identity in reproductive decision making (Fournier et al. 2023; Stock et al. 2023). Participants described how their own lived experiences shaped their views about parenthood, particularly in relation to concerns about stigma, social treatment, and the potential experiences of a future child. These findings extend existing research by demonstrating that, for individuals with appearance-related conditions, reproductive decision making is not only influenced by medical considerations and genetic risk but also by the social consequences associated with visible difference. While previous studies have identified the significance of identity and life experience in reproductive decisions (Fournier et al. 2023; Stock et al. 2023), less attention has been given to the role of societal responses and experiences of stigma in this process. The study findings contribute to a more nuanced understanding of reproductive decision making and suggest that discussions within genetic counselling may benefit from greater recognition of the social and interpersonal contexts in which these decisions are made.

Parents initially described emotional responses such as guilt and distress about their child’s condition; however, as they adjusted to their child’s needs, they adopted more solution-focused coping strategies, in line with Lazarus and Folkman’s (1984) transactional model of stress and coping.

This shift suggests that parents actively reappraised their experiences as they gained confidence, rather than remaining fixed in their initial emotional responses. Brief solution-focused interventions have been shown to be helpful in supporting parents when used as part of parenting programmes (Zimmerman et al. 2016) and may therefore be beneficial in supporting parents as they adapt to managing their child’s care.

The current study builds upon previous research identifying participants’ past social experiences of visible difference as both a burden and a resource, resurfacing painful memories while also providing practical knowledge and resilience that supported emotional readiness for parenthood. Some participants perceived that society often holds negative views about disabled people having children, highlighting how visible difference itself can create social barriers (Swift and Bogart 2021). Viewed through a social model of disability, these findings suggest that social attitudes and stigma play an important role in reproductive decision-making, alongside medical considerations (Harcourt et al. 2021). Garel et al. (2002) also noted that perceptions of society’s limited ability to provide appropriate care for disabled children may increase the likelihood of prenatal testing and termination being discussed, raising ethical dilemmas for obstetric and midwifery professionals. This study contributes to the existing research by demonstrating how people draw on their own past experiences of stigma to interpret wider societal attitudes when making decisions about parenthood (Harcourt et al. 2021; Stock et al. 2023; Swift and Bogart 2021). Social influences on reproductive decision making were highlighted in Theme Two, ‘Social perceptions, stigma, and responsibility in reproductive decision making,’ with participants expressing a desire to protect their child from suffering and from the social pressures and judgements associated with looking different. In line with previous research, participants lived experiences influenced their reproductive decision-making (Ardouin et al. 2021; Stock and Rumsey 2015), particularly negative school experiences. Negative reactions from peers and a lack of support from teachers were often recalled and fed directly into worries that their child might face similar challenges which is consistent with previous studies (Stock and Rumsey 2015). There is considerable evidence highlighting the challenges children and adolescents may face at school when living with a visible difference (Davies et al. 2026; Soon et al. 2024). The prominence of school-based memories in participants’ accounts suggests that early social experiences may hold particular emotional salience when imagining future children. This may reflect the fact that school-age years coincide with key stages of identity development, making these experiences particularly salient in later life. Alternatively, it may be that prospective parents tend to imagine their future children when young rather than as adults, leading to a heightened focus on school experiences, or a combination of both factors.

Participants’ worries about having a child who might look different was an important consideration; however, decisions were also influenced by beliefs about termination, ART, and broader social attitudes toward difference. For some, termination was experienced as a moral conflict, as it intersected with their own sense of worth and right to exist. These findings show that decisions about having children are shaped not only by concerns about a child’s quality of life, but also by how individuals make sense of their own identity. For genetic counsellors, this highlights the importance of actively exploring identity during counselling discussions and how individuals have made sense of their condition as part of who they are. Theme two also highlighted conflicting views on testing and termination; some participants wanted children and did not view their condition as a disadvantage, while noting that social norms tended to favour natural conception over IVF-PGT. In regard to generational influences on reproductive decision-making the current study found that parental support did not necessarily make participants more likely to pursue natural conception. Parenting styles influenced how participants parented their own children, with some seeking to replicate supportive approaches they had experienced, while others aimed to create a more open dialogue where they felt their own parents had struggled.

In relation to ART, some participants viewed IVF with PGT as the only viable option to avoid passing on their condition, largely due to concerns about prenatal testing, including miscarriage risk and the potential need to make post-diagnostic decisions such as termination. However, others experienced ethical discomfort, likening IVF PGT to creating “designer babies” or screening out part of their identity. Together, these findings highlight the dual role of reproductive technologies in offering hope while also generating new forms of stigma, both from wider society and within rare disease communities themselves. Previous research has shown that PGT is generally viewed as more acceptable for severe childhood or life-threatening conditions, with far less consensus when conditions have variable expression (Hughes et al. 2021; Klitzman, 2021). The conditions represented in the current study fall into this latter category, as they can range from mild to moderate, with some cases becoming potentially life-threatening. Consistent with this literature, our findings demonstrate that reproductive decisions are made within a broader social context, shaped by concerns about how reproductive technologies influence societal views of disease and disability, as well as the individuals’ own sense of identity (Hughes et al. 2021). This highlights the importance of considering how reproductive technologies are discussed in genetic counselling and their wider implications for how disability and difference are valued.

The findings point to a need for more tailored, holistic support that combines clear medical information with practical guidance for navigating social interactions, recognising adjustment as an ongoing process rather than one limited to diagnosis (Harcourt et al. 2025). Solution-focused approaches may be helpful, alongside providing space for individuals to reflect on past experiences, particularly those from school, which can resurface when considering and becoming a parent. This extends previous research showing that parents’ positive adjustment supports children’s development and appearance-related outcomes (Feragen et al., 2022) and highlights how parents who share the condition bring lived experience that shapes emotional adjustment, coping, and parenting responses. In practice, this may involve clinicians routinely initiating conversations about appearance, social experiences, and identity, rather than waiting for patients to raise these issues themselves. Open-ended questions that explore how past experiences shape views on parenting can help counter stigma-related silence and support informed decision-making. Approaches informed by attachment theory and parent–infant bonding may be particularly valuable, as they provide a framework for promoting secure, responsive relationships and confidence in early parenting (Ardouin et al. 2021).

Peer connection was an important source of support for participants, offering something often missing in wider social conversations. Limited representation of people with visible differences having children, alongside perceived stigma, meant that talking openly about parenthood and reproductive decisions often felt difficult. Participants felt that hearing the experiences of others could help normalise anxiety and uncertainty around reproductive decision-making, raising questions about how and where peer support could be appropriately facilitated. Previous research has shown that peer support groups for parents of children with genetic conditions, delivered in person or online, can help families process diagnoses and learn from others who have navigated similar situations (Craig et al., 2023). Despite its potential benefits, peer support should not be viewed as a substitute for professional healthcare support, and further research is needed to identify the most appropriate ways to facilitate it (Plumridge et al., 2011). The findings highlight a need for practical, solution focused support discussed previously alongside peer-informed resources for those considering parenthood, with charities potentially playing a key role in sharing and disseminating this support.

### Limitations

Several limitations should be considered when interpreting the findings of the current study. As a qualitative study, the sample size was small to support in-depth exploration rather than generalisation. While appropriate for the study aims, the sample may not capture the full range of conditions and experiences of individuals with inheritable conditions that affect appearance. Participants were recruited through charities, which may have shaped the perspectives represented, as those who are engaged with charities may differ from individuals who are less connected to advocacy or peer support communities. The self-selected sample may under-represent individuals experiencing higher levels of anxiety around reproductive decision-making; however, the findings can still inform the development of accessible resources for those less likely to seek support. In addition, although the study included a range of visible conditions, a broader range of conditions may have further enriched the findings. As with qualitative research more generally, the findings are not intended to be generalisable but instead offer transferable insights that may inform understanding in similar contexts.

#### Future directions

Future research could build on the current study findings by including a broader range of conditions affecting appearance and by examining potential differences in reproductive decision-making across distinct care pathways. Greater representation of men and consideration of additional socio-cultural factors, such as religious beliefs, may further enrich understanding of how reproductive choices are shaped. Longitudinal research following individuals across different stages of decision-making could also provide valuable insight into how perspectives evolve over time and in response to changing personal and social contexts. The findings highlight a need for training that addresses the role of social stigma in reproductive decision-making, particularly as visible difference is frequently stigmatised and negative attitudes toward people who look different remain widespread (Stone, 2022).

Peer support offers potential as a source of support; however, further research is needed to determine how it can be most effectively facilitated. Charities and condition specific support organisations are well placed to facilitate peer support and can play a key role within existing care pathways. Many charities already provide opportunities for individuals and families to connect with others who share lived experience of visible difference, through online meetings or family events. Integrating signposting to these organisations within genetic counselling can help recognise peer support as a valued component of care, offering practical advice and emotional validation. In addition, psychological support that provides space to reflect on experiences of living with a visible difference may help reduce parental anxiety by addressing how parents’ own experiences, consciously or unconsciously, are projected onto their child and shape reproductive decision-making.

In conclusion, this study explores how reproductive decisions are shaped not only by clinical risk but by anticipated societal attitudes toward disability and visible difference, including concerns about stigma and negative judgement. These findings extend beyond individuals with inheritable conditions that affect appearance, contributing to wider debates about reproductive decision-making and reproductive technologies. They underscore the importance of genetic counselling and reproductive healthcare that supports non-directive, socially informed discussions attentive to stigma, identity, and lived experience alongside medical information.

## Data Availability

The data supporting the findings of this study are qualitative and contain potentially identifiable information. To protect participant confidentiality, the full dataset is not publicly available. However, de-identified excerpts may be available from the corresponding author upon reasonable request and subject to appropriate ethical considerations.
